# The PPAR-γ antagonist T007 inhibits RANKL-induced osteoclastogenesis and counteracts OVX-induced bone loss in mice

**DOI:** 10.1186/s12964-019-0442-3

**Published:** 2019-10-26

**Authors:** Xiang Li, Lei Ning, Jianjun Ma, Ziang Xie, Xiangde Zhao, Gangliang Wang, Xinyu Wan, Pengcheng Qiu, Teng Yao, Haoming Wang, Shunwu Fan, Shuanglin Wan

**Affiliations:** 1 Department of Orthopaedic Surgery, Sir Run Run Shaw Hospital, Zhejiang University School of Medicine, 3 East Qingchun Road, Hangzhou, 310016 China; 2 Key Laboratory of Musculoskeletal System Degeneration and Regeneration Translational Research of Zhejiang Province, Hangzhou, 310016 China; 3 First Clinical Medical College, Wenzhou Medical University, Wenzhou, 325035 China; 4 The Third Hospital of Hebei Medical University, Shijiazhuang, 050051 China

**Keywords:** Osteoclasts, T007, Peroxisome proliferator-activated receptor-gamma, Osteoporosis, Receptor activator of nuclear factor-κB ligand

## Abstract

**Background:**

Osteoclasts are key determinant cellular components implicated in the development and progression of disorders driven by bone damage. Herein, we studied the upshot of T007, an antagonist of peroxisome proliferator-activated receptor-gamma (PPARγ), on osteoclastogenesis using cell and animal models.

**Results:**

The in vitro assays revealed that T007 hindered the osteoclastogenesis caused by the treatment with the receptor activator of nuclear factor-κB ligand (RANKL) through inhibiting the levels of PPARγ in cells. The PPARγ siRNA partially reproduced the inhibitory action of T007. The opposite findings were produced after PPARγ overexpression. Furthermore, T007 prevented from bone loss in a mouse model of osteoporosis induced by ovariectomy (OVX). These findings implied that T007 is a potential efficient drug for the prophylaxis and cure of osteoclast-related disorders.

**Conclusions:**

Taken together, our findings demonstrated that T007 impedes osteoclastogenesis and will be useful for the therapy of bone related diseases, essentially osteoporosis**.**

## Background

Bone homeostasis is actively maintained in equilibrium by osteoclast resorption and osteoblastogenesis [1, 2]. Excessive osteoclast activity can cause bone diseases such as osteoporosis [2]. Osteoporosis is a chronic disease characterized by decreased bone mass, abnormal microstructure of bone tissue, increased bone fragility and fracture, and has become a global public health problem [3]. At present, more than 1.02 billion people worldwide suffer from osteoporosis, and this number is expected to increase to 1.36 billion by 2030 [4]. Osteoprotegerin (OPG)/nuclear factor-κB receptor activating factor (RANK)/nuclear factor-κB receptor activating factor ligand (RANKL) complex is a system that plays an important role in bone metabolism, and regulates the dynamic balance of osteoblast-mediated bone matrix synthesis and osteoclast-mediated bone resorption processes [5].

RANKL is a decisive cytokine implicated in the formation of osteoclasts, which promotes the viability and differentiation of osteoclasts [6]. The RANKL-RANK complex (RANKL receptor) leads to segregation of RANK monitoring of modulators (e.g. TRAF6 gene) and its subsequent mitogen-activated protein kinase (MAPK) activation [7, 8] which upregulates the expression levels of c-Fos and nuclear factor of activated T cells 1 (NFATc1) to induce osteoclastogenesis [9]. OPG is a soluble protein secreted by osteoblasts, which increases bone density and achieves its ability to inhibit bone resorption by competitively binding RANK to RANKL [10]. RANKL/OPG ratio can be used as an important indicator for bone mass and bone health [11]. Thus, signaling pathways governed by RANKL can constitute pharmacological targets for diseases involving the degradation of bone cells as supported by the effect of neutralizing RANKL antibodies, denosumab, on the cure of osteoporosis [12].

Drugs against peroxisome proliferator-activated receptor-gamma (PPARγ) have been the subject of numerous studies because of their potential to treat a panoply of malignancies such as cancer. PPARγ is paramount involved in the formation of osteoblasts [13] since its expression level is thoroughly upregulated in osteoclastogenesis [14, 15]. PPARγ is acknowledged as a pharmacological target of exogenous drugs [16] because the PPARγ specific inhibitors cause bone loss as a consequence of amplified bone resorption [17, 18]. Although reported in some current research works, the function of PPARγ in the pathophysiology osteoclasts-induced bone disorders remains unclear and has yet to be copiously elucidated.

T0070907 (T007; PubChem database SID: 53790303) has been discovered and proven as an effective and a picky competitor of PPARγ [19]. Previous in vitro studies conveyed that PPARγ is repressed by treatment with T007 [20]. Furthermore, T007 is known to possess cancer-suppressor properties [21, 22]. For example, previous showcased that T007 modulates the expression of PPARγ to hinder the viability and motility of breast cancer cell [21]. In addition, T007 was conveyed as an inhibitor of cell adhesion and invasion and an inducer of anoikis in specific hepatocellular carcinoma cell lines via PPAR pathway [23]. In view of the above-mentioned regulatory features of PPAR signaling pathway in osteoclastogenesis, we hypothesize that T007 may represent a new approach to overcome bone diseases.

Thus, our goal was to inquire the properties of T007 on osteoclasts, decode the mechanisms mediated by T007 in osteoclastogenesis and test the restorative properties of T007 on bone loss in osteoporosis using a mouse model.

## Materials and methods

### Preparation of mouse bone marrow-derived macrophages (BMM) and osteoclast refinement

Primary BMMs were obtained by isolation from the tibia and femur bone marrow extracted from female 6-week-old C57BL/6 mice. The isolated cells were seeded in α-MEM medium (Gibco-BRL, Gaithersburg, MD, USA) containing 30 ng/mL M-CSF (R&D, Minneapolis, MN, USA), 10% FBS (Gibco-BRL, Gaithersburg, MD, USA) and 1% penicillin/streptomycin (Gibco-BRL, Gaithersburg, MD, USA). The seeding was performed at 37 °C in a 5% CO_2_ chamber until 90% confluence. Next, the BMMs (8 × 10^3^ cells/well) were cultured using 96-well plates under different indicated treatment conditions with refreshment of the medium each 2 days until formation of mature osteoclasts. Subsequently, after two washes of cells using the phosphate buffered saline (PBS), cells were fixed for about 20 min in 4% paraformaldehyde and finally stained with TRAP (Sigma-Aldrich, St. Louis, MO, USA).

### Measurement of T007 cytotoxicity on BMMS

BMMs cells were culture to 96-well plates for 24 h has indicated above at an inoculum of 2 × 10^4^ cells/well in three biological replicates in presence of 30 ng/mL of M-CSF. Different concentrations (0–4.8 μM) of T007 were used for cell treatment and seeding was achieved for 48 h, 96 h in presence or absence of 30 ng/mL of M-CSF. The cytotoxic effect of T007 was assessed by the CCK-8 assay. Briefly, 10 μL of CCK-8 reagent (Dojindo Molecular Technology, Kumamoto, Japan) was supplement to every well in the 96-well plate for an additional incubation time of 2 h. The OD value was measured using the ELX800 microplate reader (Bio-Tek Instruments, Winooski, VT, USA) at a measurement wavelength of 450 nm.

### Western blotting

Each well was inoculated with 5 × 10^5^ BMMs in 6-well plates and subsequently treated using various T007 (0, 0.15, 0.3, and 0.6 μM) (Sigma Aldrich, St. Louis, MO, USA) concentrations for 2 h as the experimental group. Untreated BMMs were used as control. Cells were activated by treatment using RANKL at a dosing of 50 ng/mL for 0, 1, 3, and 5 days. To explore whether T007 impacts on the expression of NFATc1, cells were incubated with a mixture containing and M-CSF (30 ng/mL) and RANKL (50 ng/mL), and added with or without 0.6 μM T007 for 0, 1, 3, and 5 days. The total protein was isolated from cells using the radioimmunoprecipitation assay (RIPA) lysis buffer (Sigma-Aldrich) and subsequently centrifuged for 15 min at 12,000 g. Afterward, the collection of supernatants was done and followed by 10% SDS-PAGE electrophoresis and transfer of aliquots to PVDF (Bio-Rad, Hercules, CA, USA) membrane. After blocking with a solution of 5% nonfat dry milk for 1 h at ambiant temperature, the membranes were incubated with the primary antibodies overnight at 4 °C and developed with LAS-4000 Science Imaging System (Fujiflm, Tokyo, Japan) protein. Anti-NFATc1, c-Fos, Runx2, β-catenin, RANKL, GAPDH, β-tublin and β-actin antibodies were purchased from Cell Signaling Technology (Danvers, MA, USA) and antibody against PPARγ and osteoprotegerin (OPG) were purchased from Abcam (Cambridge, MA, USA).

### RNA extraction and qRT-PCR experiments

BMMs (1 × 10^5^ cells/well) were cultured using 6-well plates in presence of 30 ng/mL M-CSF, 50 ng/mL RANKL (R&D, Minneapolis, MN, USA) and 0.6 μM T007. BMMs were cultured for different time points (0, 1, 3, and 5 days) and total RNA was isolated with the help of the RNeasy Mini Kit (Qiagen, Valencia, CA, USA). The cDNA synthesis system was: 1 μg RNA, 2 μL of 5 × PrimeScript RT Master Mix (Takara Bio, Otsu, Japan), 4 μL of RNase free distilled water, total 10 μL. The SYBR Green QPCR Master Mix (Takara Bio) kit was used for the RT-PCR experiment. The instrument used was ABI Prism 7500 system (Applied Biosystems, Foster City, CA, USA). The reaction system was 5 mL SYBR Green QPCR Master Mix, 3 mL ddH_2_O, 1 mL cDNA, 10 mM each of forward and reverse primers, for a total of 10 mL. The reaction was carried out at 95 °C for 10 min, after which each cycle was reacted at 95 °C for 10 s, 60 °C for 20 s, 72 °C for 20 s, 40 cycles and finally 72 °C for 1 min. The internal reference gene selected was GAPDH. The primer sequences are shown in Table 1.

**Table 1:** Primer sequences for qRT-PCR analysis

Primer	5′-3’
GAPDH-F	ACCCAGAAGACTGTGGATGG
GAPDH-R	CACATTGGGGGTAGGAACAC
CTSK-F	CTTCCAATACGTGCAGCAGA
CTSK-R	TCTTCAGGGCTTTCTCGTTC
c-Fos-F	CCAGTCAAGAGCATCAGCAA
c-Fos-R	AAGTAGTGCAGCCCGGAGTA
NFATc1-F	CCGTTGCTTCCAAAAATAACA
NFATc1-R	TGTGGGATGTGAACTCGGAA
Atp6v0d2-F	GACCCTGTGGCACTTTTTGTATTC
Atp6v0d2-R	GCTTGCATTTGGGGAATCTATC
DC-STAMP-F	AAAACCCTTGGGCTGTTCTT
DC-STAMP-R	AATCATGGACGACTCCTTGG
ALP-F	CCAACTCTTTTGTGCCAGAGA
ALP-R	GGCTACATTGGTGTTGAGCTTTT
OCN-F	GAGGGCAATAAGGTAGTGAACAGA
OCN-R	AAGCCATACTGGTTTGATAGCTCG
Runx2-F	TTCTCCAACCCACGAATGCAC
Runx2-R	CAGGTACGTGTGGTAGTGAGT
Acp5-F	TCCGTGCTCGGCGATGGACCAGA
Acp5-R	CTGGAGTGCACGATGCCAGCGACA
OSX-F	GCTGCAAGCTCTCCATAACC
OSX-R	GCCAGAAGCTGTGAAACCTC
COL1a-F	TCAGACCACGGACGCCATCT
COL1a-R	CGGCAACGATGGTGCTAAGG
OPG-F	TGGAGAGGTAGAAAAGGCACA
OPG-R	CAAACACACTCTTGGCACCAC
RANKL-F	GCCAGTGGGAGATGTTAG
RANKL-R	TTAGCTGCAAGTTTTCCC

### CCK-8 assay

The CCK-8 assay was the one employed for testing the cytotoxicity of T007 on BMMS. BMMs cells were plated to 96 wells at a density of 2 × 10^4^/well, three biological replicates, and 30 ng/mL of M-CSF was added for 24 h. The cells were incubated with diverse doses of T007 and added with 30 ng/mL of M-CSF for 48 h and 96 h. Next, 10 μL of CCK-8 reagent was supplemented to every well, and the 96-well plate was incubated for an additional 2 h. The OD value was measured on a microplate reader (Bio-Tek Instruments, Winooski, VT, USA) instrument using a wavelength of 450 nm.

### CHIP assay

The kit was selected from the SimpleChIP Enzymatic Chromatin IP Kit (Agarose Beads) (CST, Cambridge, MA, USA). DNAs were extracted from the treated RAW264.7 cells, and NFATc1 and genes were detected using specific primers.

### Cell transfection

Control vector (pEX-3 (pGCMV/MCS/Neo) plasmid), PPARγ vector (recombinant plasmid containing PPARγ, pEX-3 (pGCMV/MCS/Neo)-PPARγ), control siRNA and PPARγ siRNA were procured from GenePharma (Shanghai, China). The transfection kit was Lipofectamine 3000 (Invitrogen, Carlsbad, CA, USA). The vectors and siRNAs sequences were separately transfected into BMMs cells which were subsequently induced into osteoclasts. The siRNA sequences were designed and synthesized by GenePharma (Shanghai, China). The relevant sequences are as follows: PPARγ siRNA-1356: 5′-GACAGUGACUUGGCUAUAUTT-3′; PPARγ siRNA-918: 5′-GCGAUCUUGACAGGAAAGATT-3′; negative control (NC) siRNA: 5′-AUAUAGCCAAGUCACUGUCTT-3′.

### Differentiation of osteoblast stromal cells

Mouse mesenchymal stem cells MC3T3-E1 were cultured for a period of 7 days in T007-containing a-MEM, 50 mg / mL-1 ascorbic acid (Sigma-Aldrich, St. Louis, MO, USA) and 10 mM b-glycerophosphate (Sigma-Aldrich, St. Louis, MO, USA) as indicated or 21 days. The medium was revitalized every additional day. Following, the cells were stained with BCIP / NBT kit (CWBIO, Beijing, China) to detect ALP and 2% Alizarin Red S solution (pH 4.1) (Sigma-Aldrich, St. Louis, MO, USA) to visualize calcium deposition in the extracellular matrix. Mineralization was quantitated via measurements of the absorbance of the dye at 562 nm on the microplate reader after decolorization with ethyl pyridine chloride (Wako Pure Chemical Industries Ltd., Osaka, Japan).

### OVX-induced bone loss model

The animal studies were performed in corroboration with the guidelines and protocols of the National Institutes of Health (NIH) for the Care and Use of Laboratory Animals and the guidelines for the animal treatment of Sir Run Run Shaw Hospital (Zhejiang University affiliated, Hangzhou, Zhejiang). Twenty 12-weeks old female C57BL/6 mice were sedated using 3 mg/mL of pentobarbital. Subsequently, two-sided ovariectomy (OVX) of sham operation was performed. To explore therapeutic effect of T007 on osteoporosis, these mice were arbitrarily separated into four sets (*n* = 5): sham (blank control), vehicle control, low-dose T007 (0.5 mg/kg), and high-dose T007 (2.5 mg/kg). All mice were intraperitoneally injected with T007 each additional day for a period of 6 weeks in terms of which mice were sacrificed. Subsequently, the uterus was separated and weighed to estimate the effect of OVX. The femur of mice was fixed in 4% paraformaldehyde and accompanied by the subsequent histological and micro-CT analyses. The remaining femur samples from every mouse was kept in liquid nitrogen awaiting qRT-PCR and western blotting experiments.

### Micro-CT scanning

A high-resolution micro-CT scanner (SkyScan 1072; Bruker microCT, Kontich, Belgium) was utilized for the analysis of the fixed tibias. The parameters were set as follows: isometric resolution was 9 mm, X-ray energy was 80 kV and 80 mA. The reconstruction program (Skyscan) was used to detect the BV/TV, BS/BV, Tb. N, Tb. Th, Tb. Sp, and porosity.

### Histological analysis

The samples were examined under a high-quality microscope and photographed. The frequency of osteoblasts and TRAP-positive multinucleated osteoclasts (Oc.S/BS) and erosion surface (ES/BS) per field was examined in each sample.

### Statistical analysis

The results were expressed as mean ± SD. Data was analyzed using Prism 7 for Windows (GraphPad Software Inc., San Diego, CA, USA). Student’s t test was performed for intergroup comparisons. Comparisons between multiple groups were analyzed using one-way ANOVA or two-way ANOVA. *P* < 0.05 implied a significance in the differences observed between groups.

## Results

### T007 inhibited RANKL-induced osteoclastogenesis in a time- and dose-dependent manner without cytotoxic effects in vitro

**Fig. 1:**
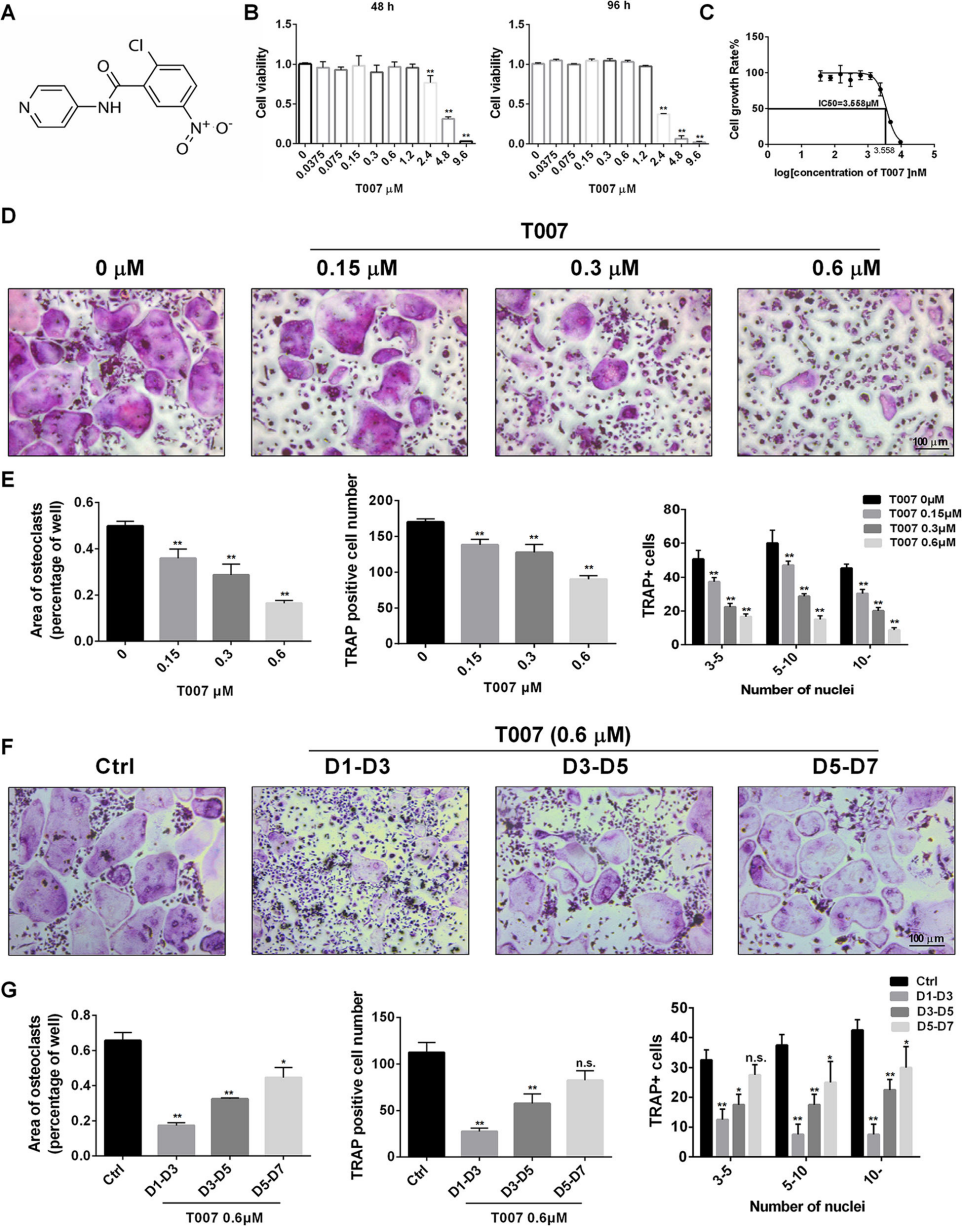
T007 inhibited RANKL-induced osteoclastogenesis in a time- and dose-dependent manner without cytotoxic effects in vitro. **a** The structure of T007. **b** Effects of T007 on BMMs viability by CCK8 assays at 48 and 96 h. **c** IC50 values obtained for the activity of T007 against BMMs. **d** BMMs were treated with various concentrations of T007, M-CSF (25 ng/mL) and RANKL (50 ng/mL) for 7 days. **e** The number, area and size of TRAP-positive multinuclear cells. **f** TRAP-positive BMMs following the treatment with 0.6 μM T007 for the indicated days during osteoclastogenesis. **g** Quantification of TRAP-positive multinuclear cells, area of osteoclasts, osteoclast number and size. All experiments were performed at least three times. Scale bar, 100 μm. *P < 0.05 and **P < 0.01 compared with the control group

In order to check the action of T007 on RANKL-induced osteoclastogenesis in vitro, we first assessed the cytotoxicity of various T007 concentrations on bone marrow-derived macrophages (BMM) cells for 48 h and 96 h. As shown in Fig. 1b, T007 concentrations greater than 2.4 μM significantly inhibited cell viability. The determination of the half maximal inhibitory concentration (IC50) indicated that the IC50 of T007 is approximately 3.558 μM (Fig. 1c). Thus, for subsequent experiments, we chose T007 concentrations of 0, 0.15, 0.3 and 0.6 μM for the dosing of BMM for 7 days. TRAP staining demonstrated that at a concentration of 0.15 μM, T007 exerted a considerable inhibition on the formation of osteoclasts. At a concentration of 0.6 μM only a low number of osteoclasts could be identified (Fig. 1d). The number of TRAP-positive cells was decreased following the rise in T007 dose, indicating a negative correlation. In addition, the size and the number of TRAP-positive cells and that of osteoclast nuclei were remarkably decreased at the concentration of 0.6 μM relatively to the control cells (Fig. 1e). To better understand the inhibitory effect of T007 during osteoclast differentiation, we treated RANKL-induced osteoclasts with a T007 concentration of 0.6 μM at different time intervals during osteoclast formation. Figure 1f and g depicted that osteoclast formation was significantly reduced after the addition of T007 at the primary phase of differentiation (days 1 to 3). Moreover, the addition of T007 at the intermediate stage of osteoclast differentiation (days 3 to 5) was also followed by a notable lessening in the amount of mature osteoclasts compared to the control group, but the degree of reduction was lower than that observed at the beginning of the differentiation. The addition of T007 at the advanced phase of osteoclastogenesis (days 5–7) was not followed by a significant decline in the amount of osteoclasts; however, small osteoclasts could be observed (Fig. 1f, g). Therefore, these observations indicate that T007 primarily hinders the osteoclastogenesis at the initial steps of osteoclast differentiation.

### T007 inhibited osteoclastic bone resorption and RANKL-induced osteoclast-specific gene expression in vitro

**Fig. 2:**
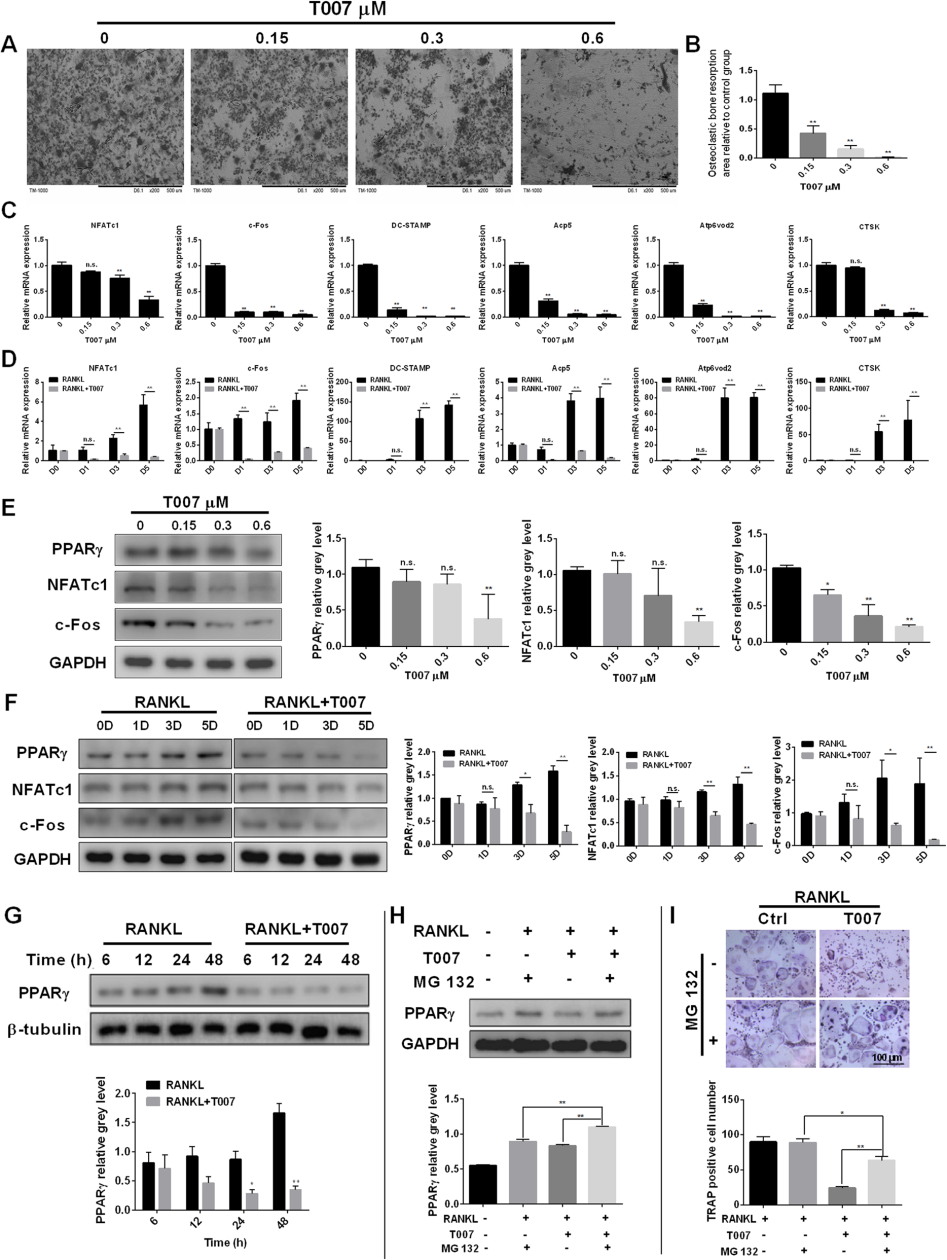
T007 inhibited osteoclastic bone resorption and RANKL-induced osteoclast-specific gene expression in vitro. **a** BMMs were seeded onto bone slices and treated the same as described in Fig. 1d for 7 days and scanning electron microscope (SEM) images of bone resorption pits are shown. **b** Resorption pit area measurements by using Image J. **c** NFATc1, c-Fos, DC-STAMP, Acp5, Atp6v0d2, and CTSK expression in BMMs treated with the indicated T007 concentrations for 5 days. **d** NFATc1, c-Fos, DC-STAMP, Acp5, Atp6v0d2, and CTSK expression in BMMs treated with T007 (0.6 μM) for 0, 1, 3 or 5 days. **e** BMMs were treated with various concentrations of T007. Cell lysates were analyzed using western blotting. The expression of PPARγ, NFATc1, and c-Fos was evaluated. **f, g** BMMs were treated with RANKL, with or without 0.6 μM T007 for the indicated periods. Cell lysates were analyzed using western blotting. The expression of PPARγ, NFATc1, and c-Fos was evaluated. **h** The effect of T007 on the expression of PPARγ in BMMs was rescued treated with 1 μM of MG 132. **i** The number of TRAP-positive BMMs after treatment with RANKL, T007 (0.6 μM), and MG 132 (1 μM) for 7 days. All experiments were performed at least three times. *P < 0.05 and **P < 0.01 compared with the control group

For a better insight into the effect of T007 on the bone resorption ability of osteoclasts, we added T007 in the medium after placing BMMs on 96-well plate bovine bone slices. The increase in T007 concentration was followed by significantly decreased area of bone resorption, especially at a concentration of 0.6 μM (Fig. 2a, b). To better figure out the hindrance of T007 on osteoclastogenesis, we measured the transcription levels of osteoclast-related genes such as DC-STAMP, CTSK, c-Fos and NFATc1. The expression of these genes was pointedly subdued by the adjunction of T007 compared with the control (Fig. 2c). Without treatment with T007, induction with RANKL promoted the expression of c-Fos, NFATc1, Acp5, DC-STAMP, CTSK and Atp6v0d2 genes. On the contrary, the concomitant application of T007 and RANKL significantly downregulated these six genes (Fig. 2d). Western blotting also indicated that the addition of different concentrations of T007 repressed the levels of PPARγ, NFATc1 and c-Fos at protein level. Specifically, 0.6 μM T007 significantly and strongly downgraded the expression of the PPARγ, NFATc1 and c-Fos (Fig. 2e). Therefore, we used 0.6 μM T007 for additional western blotting experiments. The treatment of BMMs with RANKL favored the expression of PPAR-γ, NFATc1 and c-Fos proteins in a time-dependent manner. The simultaneous treatment with both RANKL and 0.6 μM T007 inhibited PPARγ, NFATc1, and c-Fos (Fig. 2f, g). Moreover, we further found that 1 μM of MG 132, a protease inhibitor, significantly increased the expression of PPARγ (Fig. 2h) and the number of TRAP-positive cells (Fig. 2i), suggesting that MG 132 can attenuate the inhibitory effect of T007 on osteoclasts. Concurrently, we also performed an immunofluorescence analysis of PPARγ and c-Fos and found that, relatively to the control group, PPARγ and c-Fos protein expression levels were significantly reduced after the addition of 0.6 μM T007, consistent with the above results (Additional file 1: Figure S1A). To investigate whether T007 causes apoptosis of osteoclasts, we performed a flow cytometry experiment and found that when T007 was added, the number of viable cells in osteoclasts was drastically reduced and that the number of apoptotic and dead cells was significantly increased (Additional file 1: Figure S1B). To uncover the mutual interaction of proteins and genes, we performed CHIP experiments and found that T007 could block the binding of RANKL to the c-Fos gene. Thus, T007 downregulates c-Fos by blocking the interaction of RANKL to the c-Fos gene, thereby inhibiting RANKL-induced osteoclastogenesis in vitro (Additional file 1: Figure S1C). The formation of F-actin ring was also markedly decreased by 0.6 μM T007 compared with the control group (Additional file 1: Fig. S1D, E). Furthermore, PPARγ accumulated in close proximity to F-actin ring in mature osteoclast (Additional file 1: Figure S1F). It was reasonable to anticipate that PPARγ was implicated in osteoclastic bone resorption because extensive reports argued that F-actin is vital for osteoclast function [24]. Additionally, IP assay also verified that T007 can reduce the expression of PPARγ induced by RANKL (Additional file 1: Fig. S1G).

### Loss of function by knocking down PPARγ impaired RANKL-induced osteoclast-specific gene expression and inhibited osteoclast differentiation in vitro

**Fig. 3:**
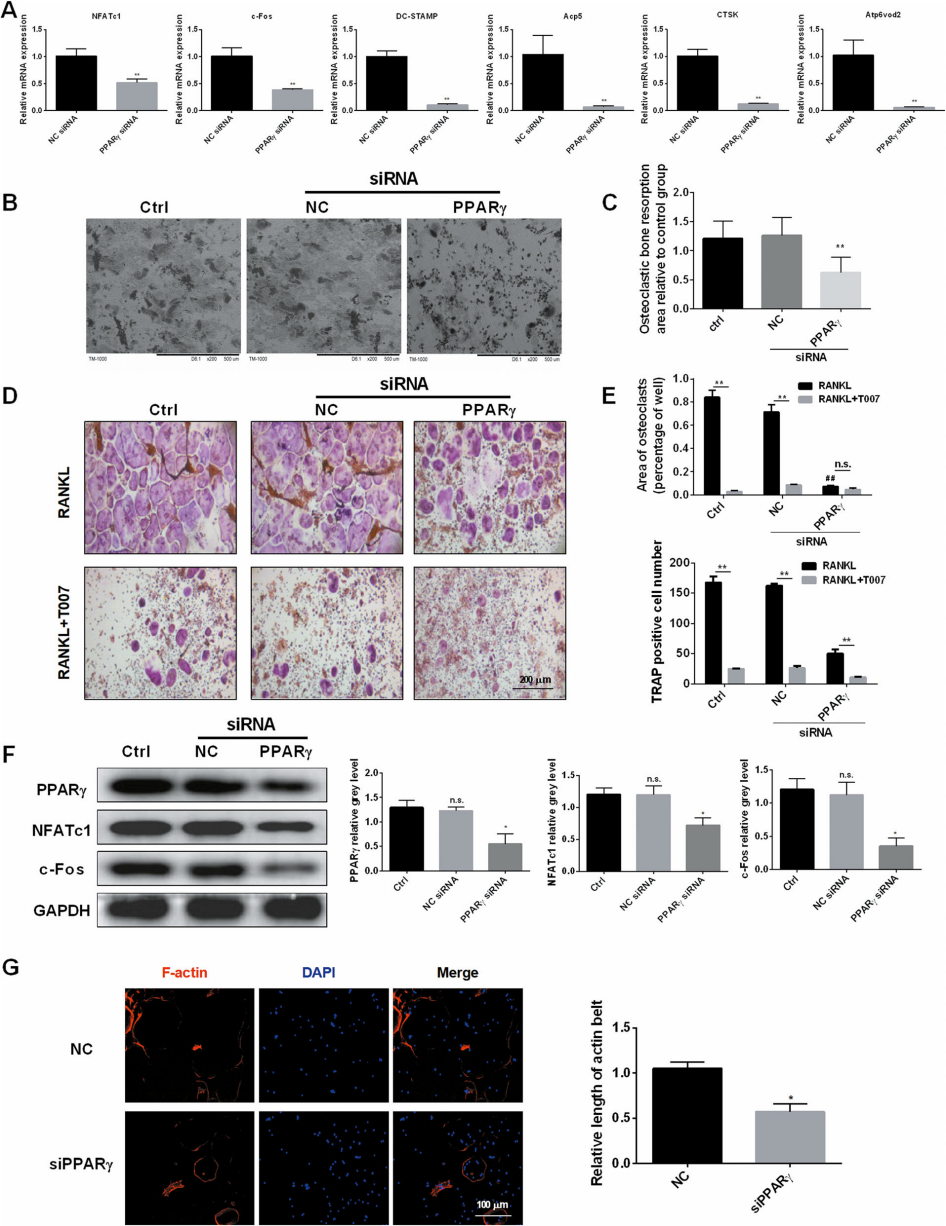
Loss of function by knocking down PPARγ impaired RANKL-induced osteoclast-specific gene expression and inhibited osteoclast differentiation in vitro. **a** Expression of the osteoclast-specific genes NFATc1, c-Fos, DC-STAMP, Acp5, Atp6v0d2, CTSK in RAW264.7 cells following the transfection with PPARγ siRNA. Negative siRNA was used as a control. **b, c** The bone resorption pits of BMMs after the transfection with siRNA were detected using scanning electron microscope (SEM) images, and the resorption pit area measurements by using Image J. Untreated cells were used as a control. **d, e** The number and area of TRAP-positive BMMs after the transfection with siRNA and the treatment with T007 (0.6 μM). Untreated cells were used as a control. **f** BMMs were transfected with siRNA, untreated cells were used as a control. Cell lysates were analyzed using western blotting. The expression of PPARγ, NFATc1 and c-Fos was evaluated. **g** The effect of siPPARγ on the formation of F-actin ring. All experiments were performed at least three times. *P < 0.05 and **P < 0.01 compared with the control group. # # *P* < 0.01 compared with the RANKL group

Next, we transfected RAW264.7 cells with PPAR-γ siRNA to study the effects of c-Fos, NFATc1, Acp5, DC-STAMP, CTSK, Atp6v0d2 gene expression. The levels of these osteoclast-specific genes were significantly downgraded when contrasted with the control group (Fig. 3a). This indicated that PPAR γ promotes the expression of osteoclast-specific genes such as c-Fos, NFATc1, and Acp5, which promotes the osteoclastogenesis. For a further understanding on the action of PPAR-γ siRNA on the bone resorption ability of osteoclasts, we detected the bone resorption pits of BMMs after the transfection with PPAR-γ siRNA. PPAR-γ siRNA significantly declined the area of bone resorption compared with the control group (Fig. 3b, c). TRAP staining displayed that, in the case of RANKL alone, the area of TRAP-positive cells and osteoclasts did not change significantly in the blank control and the negative control, but significantly decreased in the experimental group compared to the former two groups. Additionally, the concurrent treatment with RANKL and T007 decreased the area of TRAP stained cells and osteoclasts in the three groups comparatively to the case of RANKL only treatment (Fig. 3d, e). Similarly, western blot experiments showed that the expressions of PPARγ, NFATc1, and c-Fos were insignificantly different between the blank group and the negative control group, but significantly decreased in the experimental group (Fig. 3f). Moreover, siPPARγ considerably repressed the expression of F-actin compared to the control group (Fig. 3g), which was similar to the effect of T007.

### PPARγ overexpression effectively promoted the RANKL-mediated induction of osteoclast genes and osteoclast differentiation in vitro

**Fig. 4:**
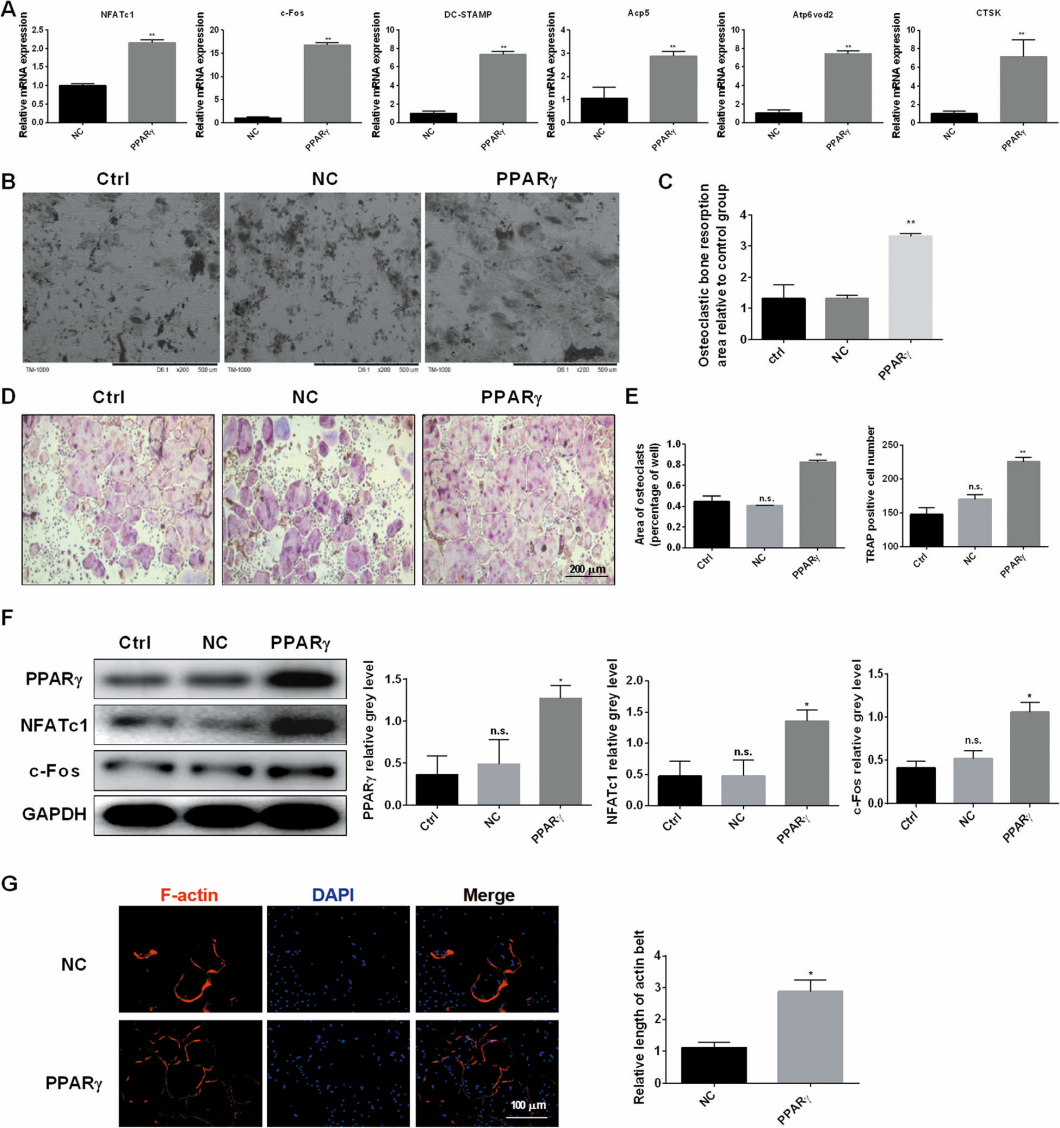
PPARγ overexpression effectively promoted the RANKL-mediated induction of osteoclast genes and osteoclast differentiation in vitro. **a** Expression of the osteoclast-specific genes NFATc1, c-Fos, DC-STAMP, Acp5, Atp6v0d2, and CTSK in RAW264.7 cells following the transfection with pcDNA-PPAR γ or the empty vector. **b, c** The effects of pcDNA-PPARγ or the empty vector on the bone resorption pits were detected using scanning electron microscope (SEM) images, and the resorption pit area measurements by using Image J. **d, e** The number and areas of TRAP-positive BMMs after the transfection with pcDNA-PPARγ or the empty vector. Untreated cells were used as a control. **f** RAW264.7 cells were transfected with pcDNA-PPARγ or the empty vector, untreated cells were used as a control. Cell lysates were analyzed using western blotting. The expression of PPARγ, NFATc1, and c-Fos was evaluated. **g** The effect of PPARγ overexpression on the formation of F-actin ring. All experiments were performed at least three times. *P < 0.05 and **P < 0.01 compared with the control group

To increase persuasiveness, we performed a reverse validation experiment that overexpressed PPARγ in RAW264.7 cells. As a result, as shown in Fig. 4a, the amount of osteoclast-specific gene expression was considerably upgraded in the experimental group transfected with pcDNA-PPARγ in contrast with the control group transfected with empty vector. Moreover, compared to the control group, PPARγ overexpression significantly increased bone resorption area (Fig. 4b, c). TRAP staining experiments showed that the frequency of TRAP-positive cells or osteoclasts in the experimental group was significantly higher compared with the blank and control groups, and there was no obvious discrepancy between the blank and the control groups (Fig. 4d, e). In western blotting, we revealed that PPARγ, NFATc1, and c-Fos protein synthesis were significantly upregulated in the experimental group (Fig. 4f). Furthermore, PPARγ overexpression significantly promoted the expression of F-actin relatively to the control group (Fig. 4g).

### T007 effected on osteoblastogenesis and osteoblast-specific gene expression

**Fig. 5:**
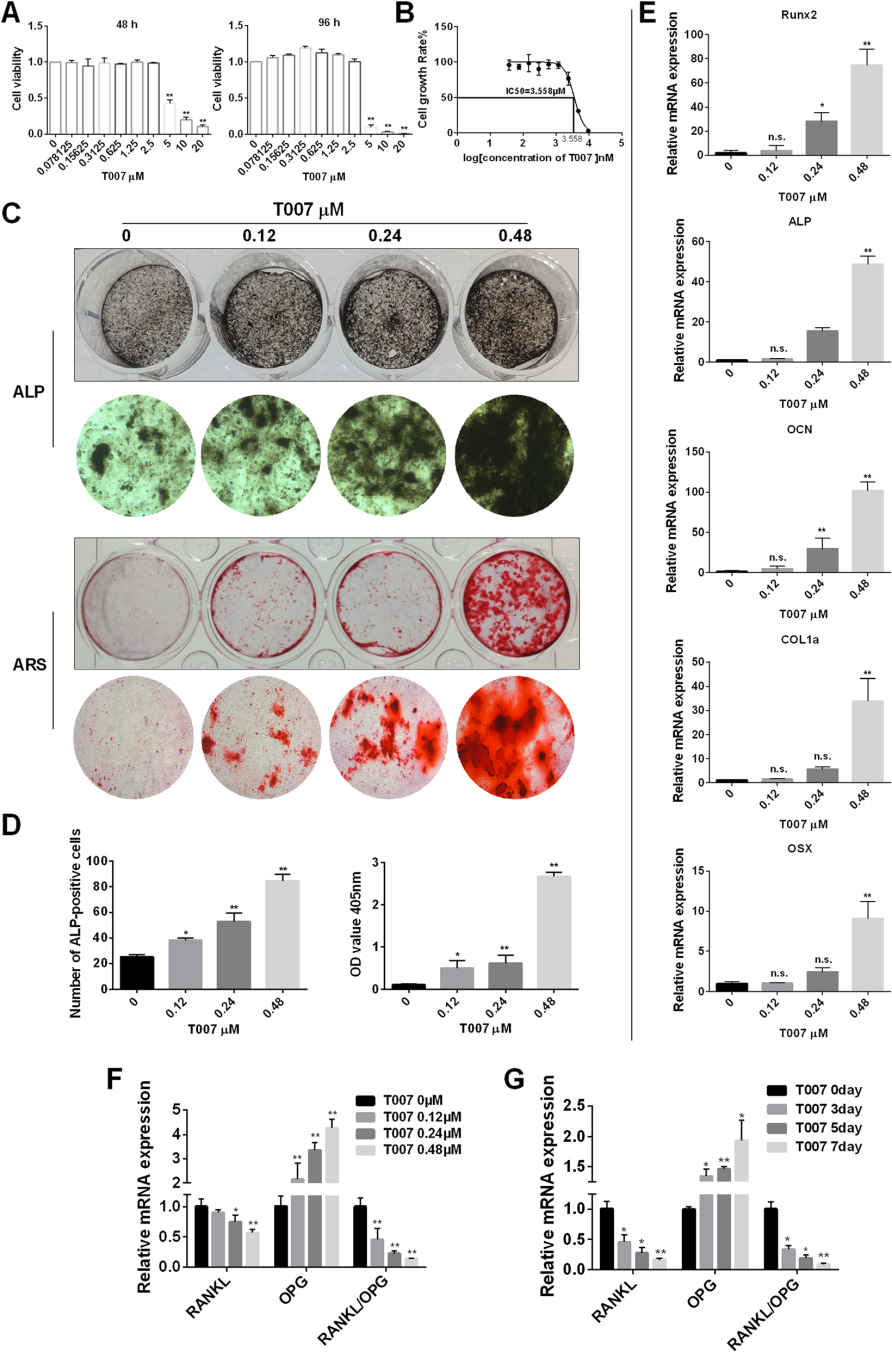
T007 effected on osteoblastogenesis and osteoblast-specific gene expression. **a** IC50 values obtained for the activity of T007 against MC3T3-E1 cells. **b** Effect of T007 on MC3T3-E1 on cell viability by CCK8 assays at 48 and 96 h. **c** ALP expression in osteoblasts and treated with various concentrations of T007 for 7 days. Mineralized extracellular matrix was detected using Alizarin Red S staining for 21 days. **d** The number of ALP-positive cells and the OD values obtained for mineralized matrix solutions following the treatment with T007. **e** Expression of the osteoblast-specific genes Runx2, ALP, OCN, COL1a, and OSX in MC3T3-E1 cells treated with various concentrations of T007 for 7 days. **f, g** The expression levels of RANKL, OPG, and RANKL/OPG in MC3T3-E1 cells treated with various concentrations of T007 for 0, 1, 3 or 5 days. The results were normalized to the expression levels of GAPDH. All experiments were performed at least three times. *P < 0.05 and **P < 0.01 compared with the control group

To explore the effects of T007 on osteoblast formation and specific gene expression in osteoblasts, we performed the following experiments. First, we treated MC3T3-E1 cells with various doses of T007. When the concentration was less than 3 μM, T007 did not affect osteoblasts. At concentrations higher than 3 μM, the cytotoxicity increased with the increase of T007 concentration with the IC50 value of 3.705 μM (Fig. 5a, b). We then chose the T007 concentrations of 0.12 μM, 0.24 μM, and 0.48 μM to treat osteoblasts, and stained the treated osteoblasts with ALP and ARS. As a result, T007-treated osteoblasts had a deep staining effect and the darkest color at 0.48 μM, indicating that T007 can promote the formation of osteoblasts in vitro (Fig. 5c, d). The treated osteoblasts were tested by qRT-PCR to gage the expression changes of osteoblast-specific markers. The expression of osteoblast-specific genes was significantly increased by 0.48 μM T007, indicating that T007 upregulated the expression levels of osteoblast-specific genes (Fig. 5e). Furthermore, we detected the effect of T007 on the expression levels of RANKL and OPG, and the results showed that T007 inhibited the expression of RANKL, promoted the expression of OPG, and decreased the ratio of RANKL/OPG in a dose- and time-dependent manner (Fig. 5f, g).

**Fig. 6:**
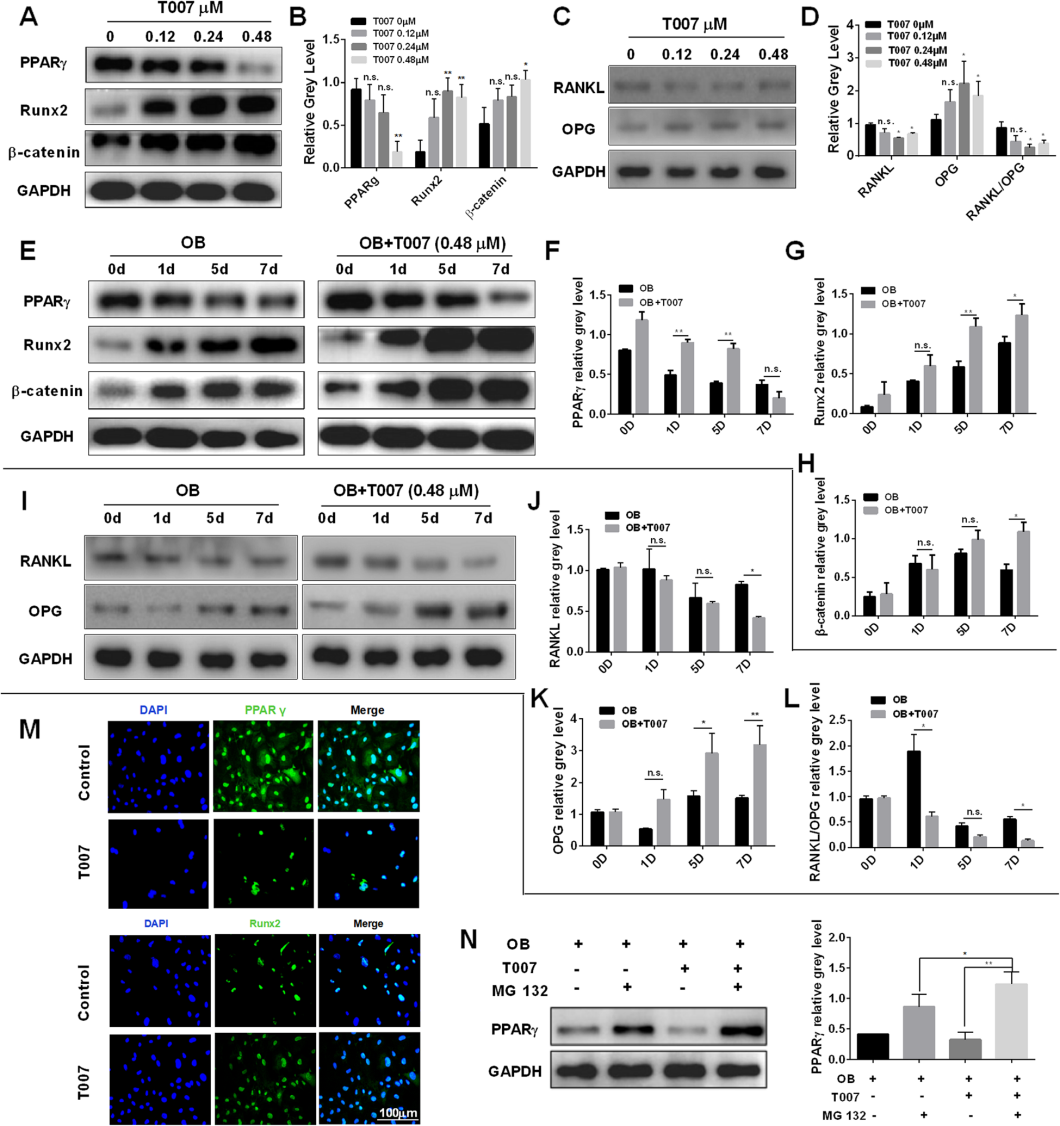
T007 promoted the expression of the osteoblast-specific genes Runx2 by inhibiting PPARγ. **a-d** MC3T3-E1 cells were treated with various concentrations of T007, and the expression of the indicated proteins was detected by western blotting. **e-l** MC3T3-E1 cells were treated with osteoblastogenic medium, and treated with 0.48 μM of T007 for the indicated day, and the expression levels of PPARγ, Runx2,β-catenin, RANKL, and OPG were evaluated by western blotting. **m** MC3T3-E1 cells were treated with or without 0.48 μM of T007. The expression levels of PPARγ and Runx2 were detected using immunofluorescence. **n** MC3T3-E1 cells were treated with osteoblastogenic medium, and treated with T007 (0.48 μM) or MG 132 (1 μM) for 7 days, and the expression of PPARγ was detected by western blotting. All experiments were performed at least three times. *P < 0.05 and **P < 0.01 compared with the control group

Western blotting was also performed to detect the expression of relevant proteins. It was found that the levels of PPARγ and RANKL and the RANKL/OPG ratio in T007-treated osteoblasts were significantly decreased, while Runx2, β-catenin and OPG were considerably upregulated (Fig. 6a-d). This implied that T007 can downregulated osteoclasts-related proteins and upregulated osteoblasts related-proteins. Next, the osteoblasts were treated with OB and PPARγ, Runx2, β-catenin, RANKL, and OPG were tested for expression levels. Compared with the control group, OB induced Runx2, β-catenin and OPG expressions but inhibited the expression of PPARγ and RANKL and the RANKL/OPG ratio. When osteoblasts were concurrently treated with OB and 0.48 μM T007, the promoting effect was more pronounced and there was a remarkable difference compared to OB treatment alone (Fig. 6e-l). At the same time, we performed immunofluorescence analysis of PPARγ and Runx2, and found that compared with the control group, the expression of PPAR γ protein in osteoblasts was significantly decreased and Runx2 protein expression was increased after adding 0.48 μM of T007 (Fig. 6m). Furthermore, we also found that 1 μM of MG 132 significantly upregulated the expression of PPARγ (Fig. 6n), indicating that MG 132 can attenuate the promotion effect of T007 on osteoblasts.

### Knocking down PPARγ promotes osteoblastogenesis and the expression of osteoblast-specific gene expression in vitro

**Fig. 7:**
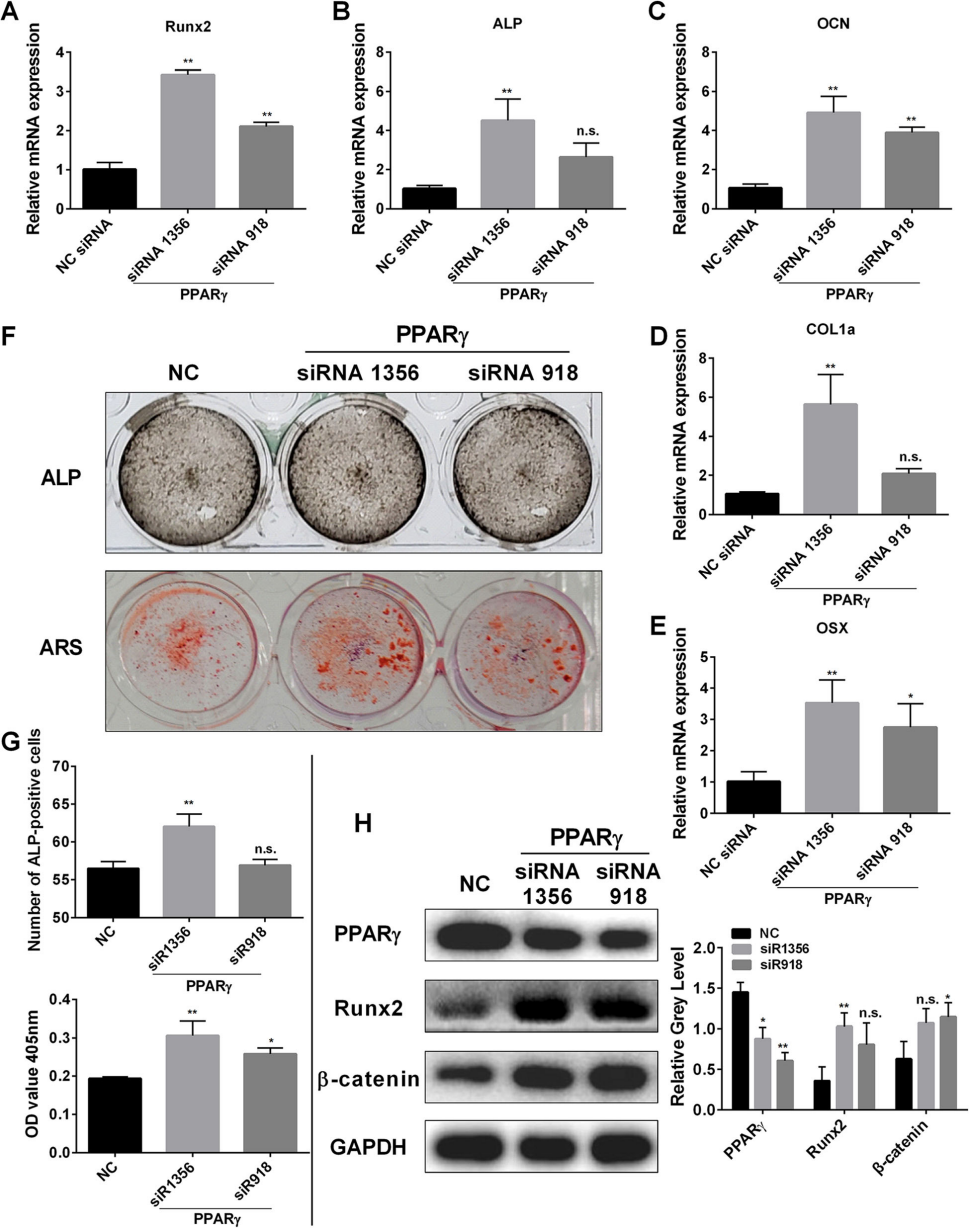
Knock-down of PPARγ promotes osteoblastogenesis and the expression of osteoblast-specific gene expression in vitro. **a-e** Expression of the osteoblast-specific genes Runx2, OCN, OSX, ALP, COL1a, and OSX in osteoblasts following the transfection with two kinds of siRNA or the negative control. **f** ALP expression in osteoblasts cultured in the osteogenic medium and mineralized extracellular matrix was detected using Alizarin Red S staining treated after transfection with two kinds of siRNA or the negative control for 7 days and 21 days. **g** The number of ALP-positive cells and the OD values obtained for mineralized matrix solutions following transfected with siRNA or the negative control. **h** Osteoblasts were transfected with two kinds of siRNA or the negative control. Cell lysates were analyzed using western blotting. The expression of PPARγ, Runx2, and β-catenin was evaluated. All experiments were performed at least three times. *P < 0.05 and **P < 0.01 compared with the control group

Osteoblasts were transfected with two siRNAs (siRNA1356, siRNA918) and the empty vector, respectively. The transfection with siRNA1356 and SiRNA918 inhibited PPARγ expression. The results showed that Runx2, OCN, ALP, COL1a and OSX genes were significantly upregulated in the two groups (Fig. 7a-e). ALP and ARS staining indicated that siRNA silencing led to darker-stained compared to the negative control group, indicating that osteoblast differentiation was promoted (Fig. 7f). The statistical results were consistent with these observations (Fig. 7g). Western blotting was also used to gage PPAR γ, Runx2, and β-catenin a protein expression levels. The results are shown in Fig. 7h and indicated that in the experimental group, the expression level of PPAR-γ protein was downregulated, while the synthesis of Runx2 and β-catenin increased was increased.

**Fig. 8:**
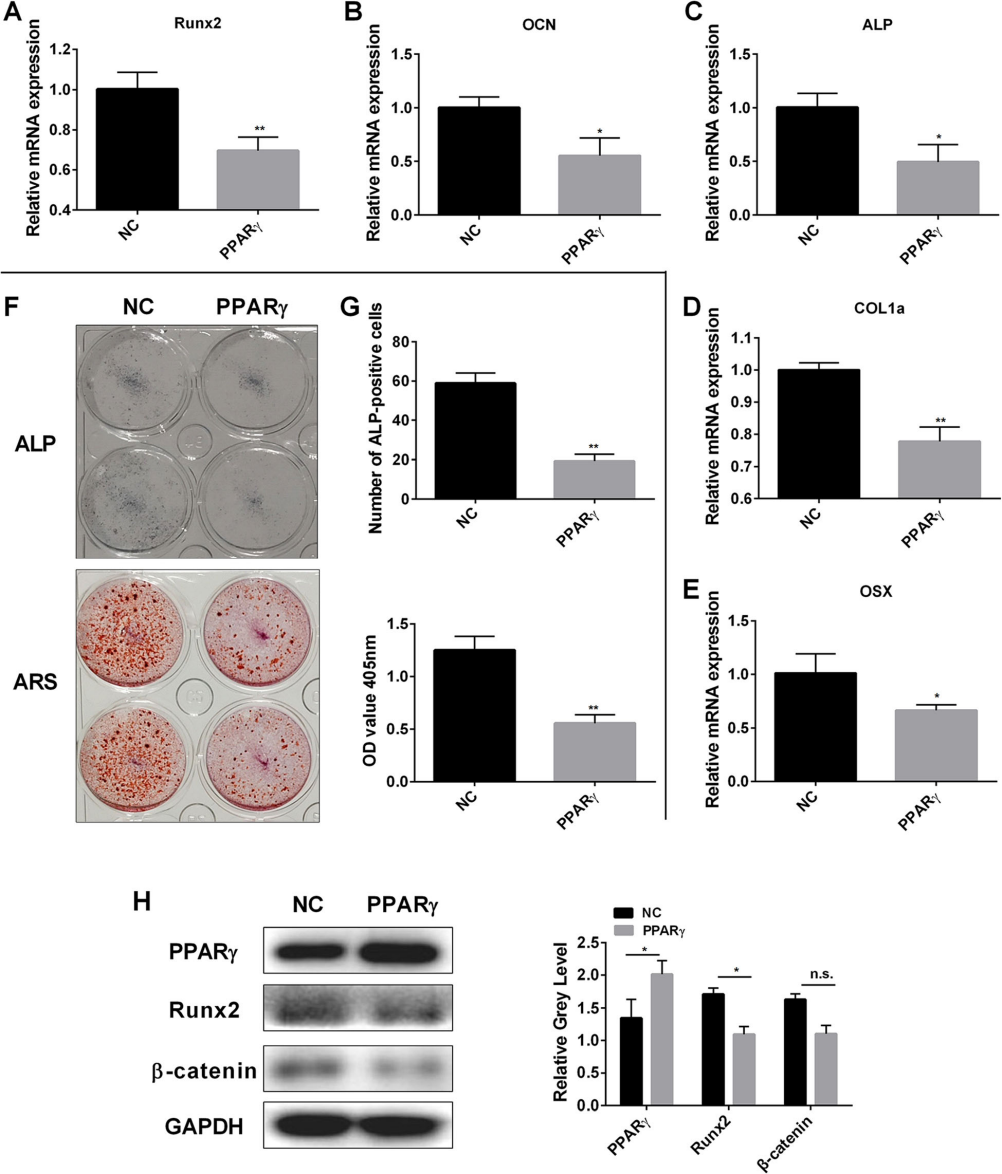
PPARγ overexpression impaired osteoblastogenesis and the expression of osteoblast-specific gene expression in vitro. **a-e** Expression of the osteoblast-related genes Runx2, OCN, ALP, COL1a, and OSX in osteoblasts following the transfection with pcDNA-PPARγ or the empty vector. **f** ALP expression in osteoblasts and mineralized extracellular matrix was detected using Alizarin Red S staining treated following the transfection with pcDNA-PPARγ or the empty vector for 7 days and 21 days. **g** The number of ALP-positive cells following the transfection with pcDNA-PPARγ or the empty vector for 7 days. **h** Osteoblasts were transfected with pcDNA-PPARγ or the empty vector. Cell lysates were analyzed using western blotting. The expression of Runx2, β-catenin, and PPARγ was evaluated. All experiments were performed at least three times. *P < 0.05 and **P < 0.01 compared with the control group

On the contrary, the overexpression of PPARγ revealed a significant decrease in the expression levels of osteoblast-specific genes Runx2, OCN, ALP, COL1a and OSX (Fig. 8a-e). The ALP, ARS staining, osteoblast staining of PPAR γ overexpression was lighter than the negative control group. The number of ALP stained cells and the OD at 405 nm decreased significantly (Fig. 8f, g). Western blotting assay showed that PPARγ, β-catenin and Runx2 were upregulated (Fig. 8h).

### T007 promotes BMSCs differentiation into osteoblasts in vitro and expression of the osteoblast-specific genes

**Fig. 9:**
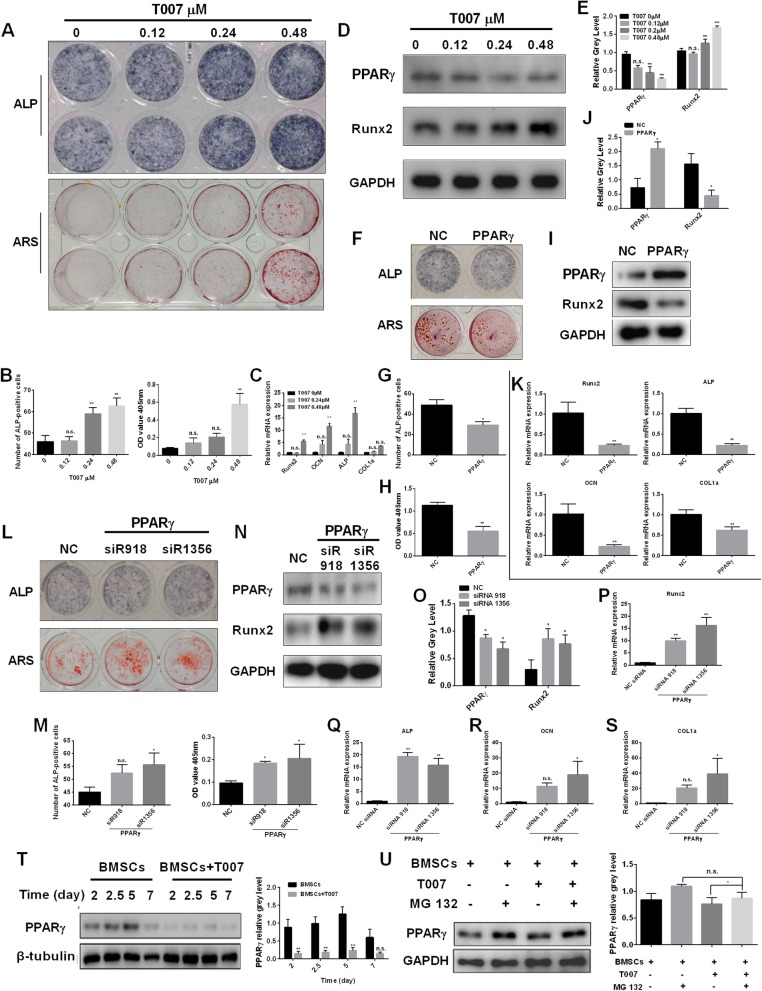
T007 promotes BMSCs differentiation into osteoblasts in vitro and expression of the osteoblast-specific genes. **a** ALP expression in BMSCs cultured in the osteogenic medium and treated with various concentrations of T007 for 7 days. Mineralized extracellular matrix was detected using Alizarin Red S staining for 21 days. **b** Expression of the osteoblast-specific genes Runx2, OCN, ALP, and COL1a in BMSCs with T007-treated maintained in osteogenic medium for 7 days. **c** The number of ALP-positive BMSCs and the OD values obtained for mineralized matrix solutions following the treatment with T007. **d, e** BMSCs were treated with various concentrations of T007, and the expression of the indicated proteins was detected by western blotting. **f** ALP expression in BMSCs and mineralized extracellular matrix was detected using Alizarin Red S staining treated following the transfection with pcDNA-PPARγ or the empty vector for 7 days and 21 days. **g, h** The number of ALP-positive cells following the transfection with pcDNA-PPARγ or the empty vector for 7 days. **i, j** BMSCs were transfected with pcDNA-PPARγ or the empty vector. Cell lysates were analyzed using western blotting. The expression of Runx2, PPARγ was evaluated. **k** Expression of the osteoblast-related genes Runx2, OCN, ALP, and COL1a in BMSCs following the transfection with pcDNA-PPARγ or the empty vector. **l** ALP expression in BMSCs cultured in the osteogenic medium and mineralized extracellular matrix was detected using Alizarin Red S staining treated after transfected with two kinds of siRNA or the negative control for 7 days and 21 days. **m** The number of ALP-positive cells and the OD values obtained for mineralized matrix solutions following transfected with siRNA or the negative control. **n, o** BMSCs were transfected with two kinds of siRNA or the negative control. Cell lysates were analyzed using western blotting. The expression of PPARγ, Runx2 was evaluated. **p-s** The expression levels of the osteoblast-specific genes (Runx2, OCN, OSX, ALP, and COL1a) in BMSCs following the transfection with two kinds of siRNA or the negative control. **t** BMSCs were treated with or without 0.48 μM of T007 for the indicated periods. Cell lysates were analyzed using western blotting. The expression of PPARγ was evaluated. **u** BMSCs were treated with T007 (0.48 μM) or MG 132 (1 μM) for 7 days, and the expression of PPARγ was detected by western blotting. All experiments were performed at least three times. *P < 0.05 and **P < 0.01 compared with the control group

To explore whether T007 affects the osteoblastogenesis of BMSCs in vitro, we added different concentrations of T007 to the osteogenic medium of BMSCs. The addition of 0.48 μM T007 led to the deepest ALP and ARS stained cells (Fig. 9a). The statistical results were coherent with these observations (Fig. 9b). Furthermore, the Runx2, OCN and ALP mRNA levels were also significantly increased while the COL1a mRNA level was increased but showed no significant difference (Fig. 9c). Western blotting indicated that, the addition of 0.48 μM T007 showed a significant downregulation of PPAR-γ protein and a significant increase in Runx2 expression (Fig. 9d, e). After transfection of BMSCs with pcDNA-PPARγ, ALP and ARS staining displayed that the amount of osteoblasts in the experimental group were lower compared to the control group (Fig. 9f-h). Western blotting indicated an increase in PPARγ protein expression and a downregulation in Runx2 expression (Fig. 9i, j). The expression levels of osteoblast-specific genes were decreased (Fig. 9k). On the contrary, the transfection of BMSCs with siR918, siR1356 led to the deepest ALP and ARS stained cells (Fig. 9l, m). In addition, the transfection of BMSCs with siR918 and siR1356 increased the number of osteoblasts with decreased PPARγ protein expression (Fig. 9n, o). The Runx2 protein expression was, on the contrary, increased (Fig. 9n, o). The levels of osteoblast-specific genes were significantly increased (Fig. 9p-s). Additionally, the treatment of BMSCs with 0.48 μM of T007 significantly inhibited the expression level of PPARγ in a time-dependent manner (Fig. 9t). T007 can reduce PPARγ expression in BMSCs, and this inhibitory effect can be attenuated by MG 132 (Fig. 9u).

### T007 prevents from OVX-induced bone loss

**Fig. 10:**
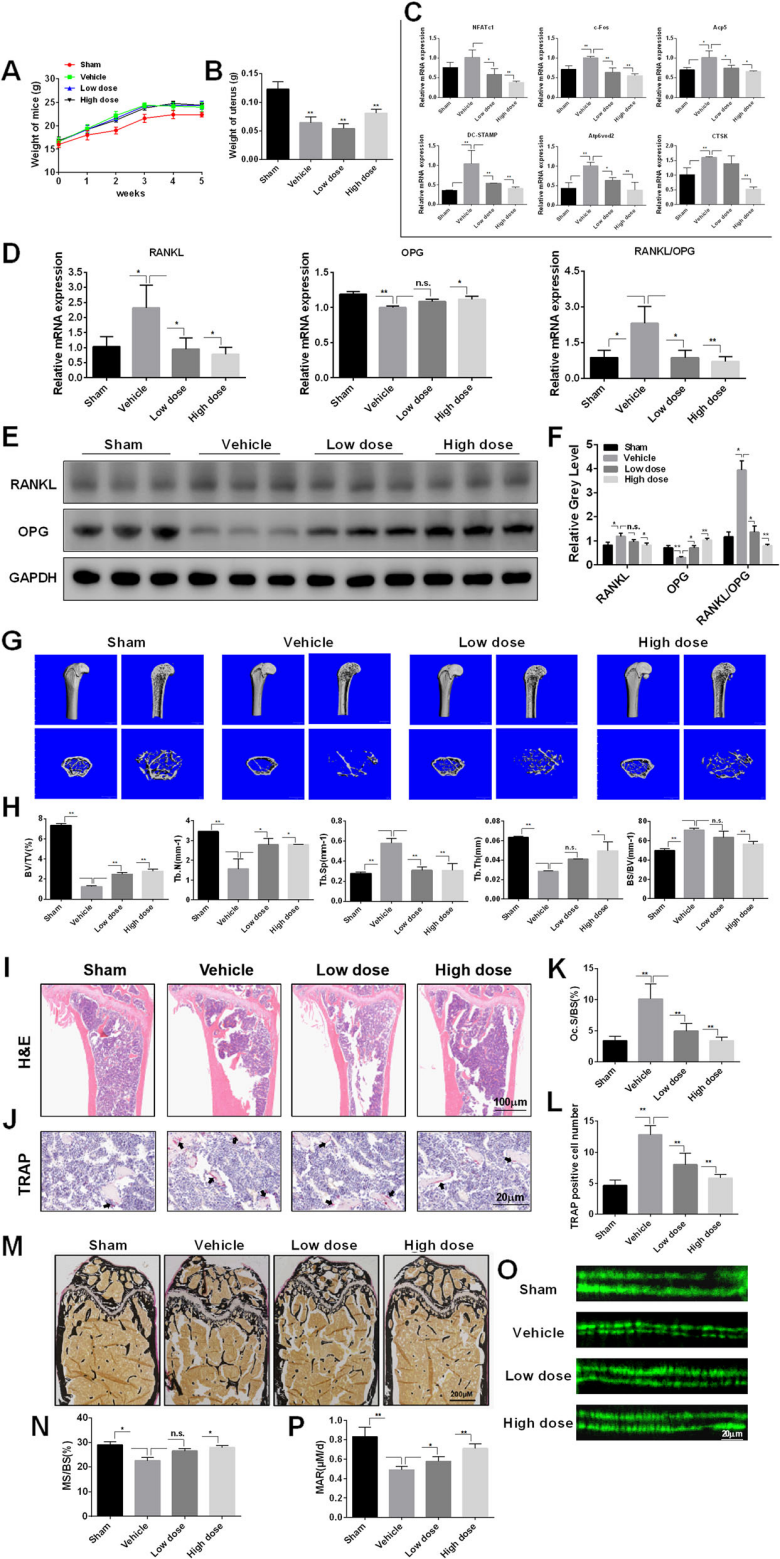
T007 prevention of OVX-induced bone loss in vivo. Verify whether the osteoporosis mouse model induced by OVX was successfully constructed through measuring mouse body weight (**a**) and uterine weight (**b**). **c** The expression levels of NFATc1, c-Fos, DC-STAMP, Acp5, Atp6vod2, CTSK were examined using qRT-PCR. The mRNA and protein expression levels of RANKL, OPG, and RANKL/OPG were detected using RT-qPCR (**d**) and western blotting (**e**, **f**), respectively. **g** The femur of each mouse were scanned with micro-CT. **h** The BV/TV, BS/BV, Tb. Sp, Tb. T and Tb. N of each sample was measured. Sections of femur were stained with H&E (**i**) and TRAP (**j**) staining. **k, l** The OcS/BS and number of osteoclasts per field of each sample was analyzed. **m, n** Sections of femurs were stained with von Kossa, and calculated the MS/BS (%). **o, p** Calcein double fluorescent labeling to observe the mouse femur MAR. All experiments were performed at least three times. *P < 0.05 and **P < 0.01 compared with the control group. BV/TV: bone volume per tissue volume, BS/BV: bone surface per bone volume, Tb.Sp: trabecular separation, Tb.T: trabecular thickness, Tb.N: trabecular number, OcS/BS: osteoclast surface per bone surface, MS/BS (%): the ration of mineralized area to bone surface area, MAR: mineral apposition rate

To validate the potential therapeutic effect of T007 on osteoporosis, we further examined T007 action in a mouse model of osteoporosis. Compared with the sham group, vehicle (OVX) treatment significantly increased the body weight and decreased the uterine weight of mice, indicating that the osteoporosis model was successfully constructed (Fig. 10a b). The qRT-PCR experiments indicated that the osteoclastogenesis related genes were promoted in animals bearing osteoporosis comparatively to the healthy animals. The treatment of mice with T007 decreased the levels of these osteoclastogenesis genes in a dose-dependent fashion compared to the osteoporosis animals administered with the vehicle (Fig. 10c). Moreover, T007 treatment significantly increased the mRNA and protein expression levels of OPG in OVX-induced bone loss models, while inhibiting RANKL expression and the ratio of RANKL/OPG (Fig. 10d-f). This implied that T007 prevents from OVX-induced bone loss. Micro-CT analysis indicated an obvious bone loss in the femur of osteoporosis animals treated with vehicle. Bone quality indices including such as BV/TV, Tb. N, and Tb. Th values were decreased while Tb. Sp and BS/BV values were significantly upgraded (Fig. 10g, h). Hematoxylin and eosin (H&E) histopathological examination revealed reduction in the number and the thickness of trabecular (Fig. 10i). Also, TRAP staining indicated that the number of multinucleated osteoclasts was obviously enlarged in the vehicle-treated animals but were decreased in the T007-treated groups (Fig. 10j). Additionally, T007 significantly reduced the osteoclast surface/bone surface area (OcS/BS) and TRAP positive cell number (Fig. 10k, l), suggesting that T007 can repress osteoclast formation and osteoclastic bone loss in vivo. The von Kossa staining results showed that the trabecular bone density and the ratio of mineralized area to bone surface area (MS/BS (%)) were declined in the vehicle-treated group, while T007 treatment could improve these results (Fig. 10m, n). Furthermore, the calcein dual fluorescent labeling revealed that compared with the vehicle group, T007 remarkably increased the MAR of the femur (Fig. 10o, p).

## Discussion

The occurrence of osteoporosis is a complex process. Previous studies have confirmed that multiple stimuli and numerous regulatory factors involved in the pathogenesis of osteoporosis interact with each other to ultimately lead to the occurrence of osteoporosis subsequently to their combined regulatory action [25, 26]. Although many therapies for osteoclasts such as estrogen-replacement therapy and bisphosphonates have been performed [27], further alternative therapies are needed due to the multiple side effects of existing therapies. Recent studies have shown that bisphosphonates have serious side effects of mandibular necrosis [28]. Here, we explored the effect of PPARγ antagonist T007 on the differentiation and resorption of multinucleated osteoclasts. In this research, we demonstrated that T007 can prevent RANKL-induced osteoclastogenesis through inhibiting PPARγ and promoting OPG to subsequently induce the osteoblast differentiation in vitro. Furthermore, our results also showed that T007 can prevent OVX-induced bone loss in vivo. Therefore, our study found that T007 is a candidate drug for treating osteoporosis, and our results help to enlighten the mechanism underlying the effect of T007, which is beneficial for further research and application.

The OPG/RANKL/RANK complex is a regulatory system closely related to the occurrence of osteoporosis and can affect the function of osteoclasts; especially, the change of OPG/RANKL ratio can directly affect the proliferation and differentiation of osteoclasts, thereby affecting bone metabolism [29, 30]. OPG competes with RANK for binding to RANKL, thereby blocking the binding of RANKL to RANK on the precursor cell membrane, delaying the differentiation of precursor osteoclasts, and reducing bone resorption. The change of OPG/RANKL ratio is the most direct indicator of the activation and proliferation of osteoclasts, which decrease will promote the proliferation and differentiation of osteoclasts, leading to the occurrence of osteoporosis [31]. Studies have found that certain Chinese medicines such as Yunnan Baiyao can upregulate OPG expression and downregulate RANKL expression in osteoblasts, which reduces the formation of osteoclasts and inhibits the activation of osteoclasts [32]. To investigate whether T007 indirectly control osteoclast differentiation, we examined the expression levels of RANKL and OPG during osteoblast differentiation, and analyzed the relative ratio of RANKL/OPG. We found that T007, an antagonism of PPARγ, significantly increased OPG expression and declined RANKL expression and RANKL/OPG ratio. Lee, Lee [33] found that rosiglitazone, an agonist of PPARγ, inhibits osteoclastogenesis through downregulating the expression of RANKL and upregulating the expression of OPG in osteoblasts. However, a recent study has shown that PPAR agonists reduced alveolar bone loss in the short term (one week), while promoted osteoclast formation by downregulating OPG expression and upregulating RANKL expression after eight weeks [34], which was similar to our results. Thus, this finding suggests that the effects of T007 on osteoclastogenesis and osteoblastogenesis are possibly driven by a regulatory mechanism involving the OPG/RANKL/RANK regulatory module.

PPARγ, a ligand-dependent nuclear receptor, regulates a variety of physiological and disease processes such as inhibiting inflammatory responses and regulating bone cell differentiation [35, 36]. Former research teams reported that PPARγ can induce osteoclastogenesis but inhibits osteoblast differentiation [37, 38]. Rzonca et al. conveyed that thiazolidinedione-activated PPARγ inhibits osteoblast differentiation [39]. In addition, PPARγ overexpression increases RANKL/OPG ratio, leading to reduced bone formation, thereby accelerating OVX-induced bone loss in female mice [40]. Jeon, Kim [41] showed that PPARγ activation can downregulate expression and function of Runx2 to inhibit osteoblast differentiation. An in vivo mouse experiment showed that PPARγ can promote osteoclast differentiation through regulating the expression of c-Fos [42]. In this study, we found that PPARγ is increased in RANKL-induced osteoclasts. Moreover, PPARγ overexpression pointedly lessened Runx2 expression level while upregulating the levels of the osteoclastogenesis regulators such as NFATc1 and c-Fos, which indicated that PPARγ is a key factor that hinders osteoblastogenesis and promotes osteoclastogenesis.

We additionally scrutinized the action of PPARγ on RANKL-induced osteoclasts and its underlying mechanisms. Antagonism of PPARγ with T007 promoted ALP expression [43]. The opposite result was obtained in the previous study of PPARγ agonists [44, 45]. Previous studies showed that the class II histone deacetylase 9 (HDAC-9) inhibits osteoclast differentiation and bone resorption by repressing PPARγ activity [46]. Similar results were found regarding the levels of factors in RANKL-induced osteoclasts. T007 decreased the expression levels of PPARγ and osteoclastogenesis-specific genes such as NFATc1, c-Fos and DC-STAMP, and promoted β-catenin and Runx2 expression levels. NFATc1 and Runx2 are both determinant transcription factors involved in the regulation of osteoclastogenesis and osteogenic differentiation [47, 48]. Runx2 was recently reported to upgrade the osteoblastogenesis of MSCs [49]. Here, Runx2 expression was increased, suggesting that T007 promotes osteoblast differentiation. NFATc1 is a major regulator of osteoclast differentiation induced by RANKL and regulates other osteoclast-specific genes including CTSK, c-Fos, Atp6v0d2 and DC-STAMP [50, 51]. In addition, previous studies indicated that DC-STAMP silencing significantly inhibited the formation of multinucleated osteoclast-like cells [52]. Our findings hinted that T007 significantly reduced expression of NFATc1 both at protein and mRNA levels. Equally, T007 downregulated the levels of CTSK, c-Fos, DC-STAMP, Acp5 and Atp6v0d2, indicating that T007 can inhibit RANKL-induced osteoclasts activity. Furthermore, we examined whether T007 could affect the OVX-induced bone loss in vivo. RT-PCR results, micro-CT, HE staining, and von Kossa staining analysis indicated T007 hinders the osteoclastic bone loss in vivo through upregulating OPG expression and downregulating RANKL expression. These results conformed with previous studies showing that metformin markedly increases tibia bone mineral density and reduces TRAP-positive cells in the OVX rats by increasing OPG expression and decreasing RANKL expression to prevent bone loss [53]. In summary, our study signposted that T007 suppresses RANKL-induced osteoclastogenesis and induces osteoblastogenesis in cell and animal studies. These findings may be beneficial for the handling of bone diseases and bone tissue engineering.

## Conclusion

As shown in Additional file 2: Figure S2, T007 reduces PPARγ expression that inhibits the differentiation of osteoclast progenitors into osteoclasts, thus attenuating bone resorption. In osteoclast progenitor, T007 suppresses c-Fos expression, which stimulates osteoclastogenesis. Conversely, T007 restrains PPARγ activation that promotes pre-osteoblasts differentiation into osteoblasts and contributes to BMSCs growth by increasing Runx2 expression, thus enhancing bone formation. Consequently, T007 prevents bone loss by tipping in balance of bone remodeling through concerned stimulation of bone resorption and inhibition of bone formation.

## Supplementary information

**Additional file 1: Figure S1.** T007 regulates the expression of PPARγ, c-Fos and F-actin, promotes the cell apoptosis and blocks the interaction between c-Fos and RANKL in vitro. BMMs were treated with or without 0.6 μM of T007. (A) The expression levels of PPARγ and c-Fos were detected using immunofluorescence. (B) The effect of T007 on cell apoptosis of osteoclasts was analyzed using flow cytometry. (C) ChIP experiment was used to detect whether T007 can block the binding of RANKL to c-Fos gene. (D, E) Representative images for F-actin ring formation using immunofluorescence. (F) The expression levels of F-actin ring and PPARγ were measured using immunofluorescence. (G) The expression of PPARγ was detected using IP assay. All experiments were performed at least three times. *P < 0.05 and **P < 0.01 compared with the control group.

**Additional file 2: Figure S2.** A simplified model of the mechanism by which T007 inhibits osteoclastogenesis and osteoblastogenesis. T007 reduces PPARγ expression that inhibits osteoclast progenitor’s differentiation into osteoclasts, thus attenuating bone resorption. In osteoclast progenitor, T007 suppress c-Fos expression, which stimulates osteoclastogenesis. Conversely, T007 restrains PPARγ activation that promotes pre-osteoblasts differentiation into osteoblasts and contributes to BMSCs growth by increasing Runx2 expression, thus enhancing bone formation. Consequently, T007 results in bone loss by tipping in balance of bone remodeling through concerned stimulation of bone resorption and inhibition of bone formation.

## Data Availability

All data generated or analyzed during this study are included in this published article.

## Abbreviations

BMM: Bone marrow-derived macrophages
MAPK: Mitogen-activated protein kinase
NFATc1: Nuclear factor of activated T cells 1
OPG: Osteoproteoprotein
OVX: Ovariectomy
PBS: Phosphate buffered saline
PPARγ: Peroxisome proliferator-activated receptor-gamma
RANKL: Receptor activator of nuclear factor-κB ligand
RIPA: Radioimmunoprecipitation assay
