# Supplementary figures and images for: The PPAR-γ antagonist T007 inhibits RANKL-induced osteoclastogenesis and counteracts OVX-induced bone loss in mice
Source: Cell Commun Signal. 2019 Oct 26;17:136. doi: 10.1186/s12964-019-0442-3 (PMC6815399; doi:10.1186/s12964-019-0442-3)

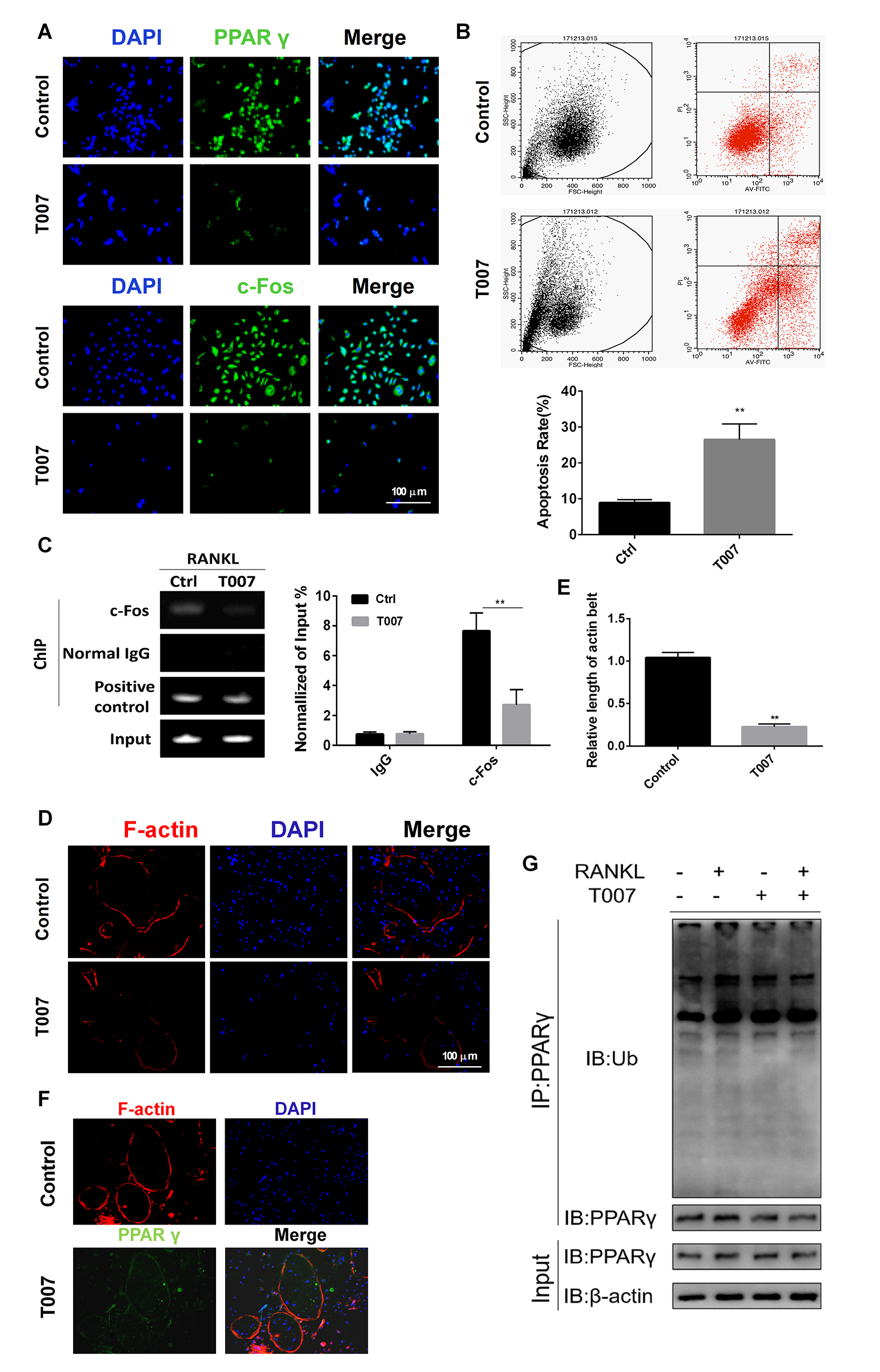

Supplement: Supplementary file 1 — Additional file 1: Figure S1. T007 regulates the expression of PPARγ, c-Fos and F-actin, promotes the cell apoptosis and blocks the interaction between c-Fos and RANKL in vitro. BMMs were treated with or without 0.6 μM of T007. (A) The expression levels of PPARγ and c-Fos were detected using immunofluorescence. (B) The effect of T007 on cell apoptosis of osteoclasts was analyzed using flow cytometry. (C) ChIP experiment was used to detect whether T007 can block the binding of RANKL to c-Fos gene. (D, E) Representative images for F-actin ring formation using immunofluorescence. (F) The expression levels of F-actin ring and PPARγ were measured using immunofluorescence. (G) The expression of PPARγ was detected using IP assay. All experiments were performed at least three times. *P < 0.05 and **P < 0.01 compared with the control group. [file 12964_2019_442_MOESM1_ESM.tif]

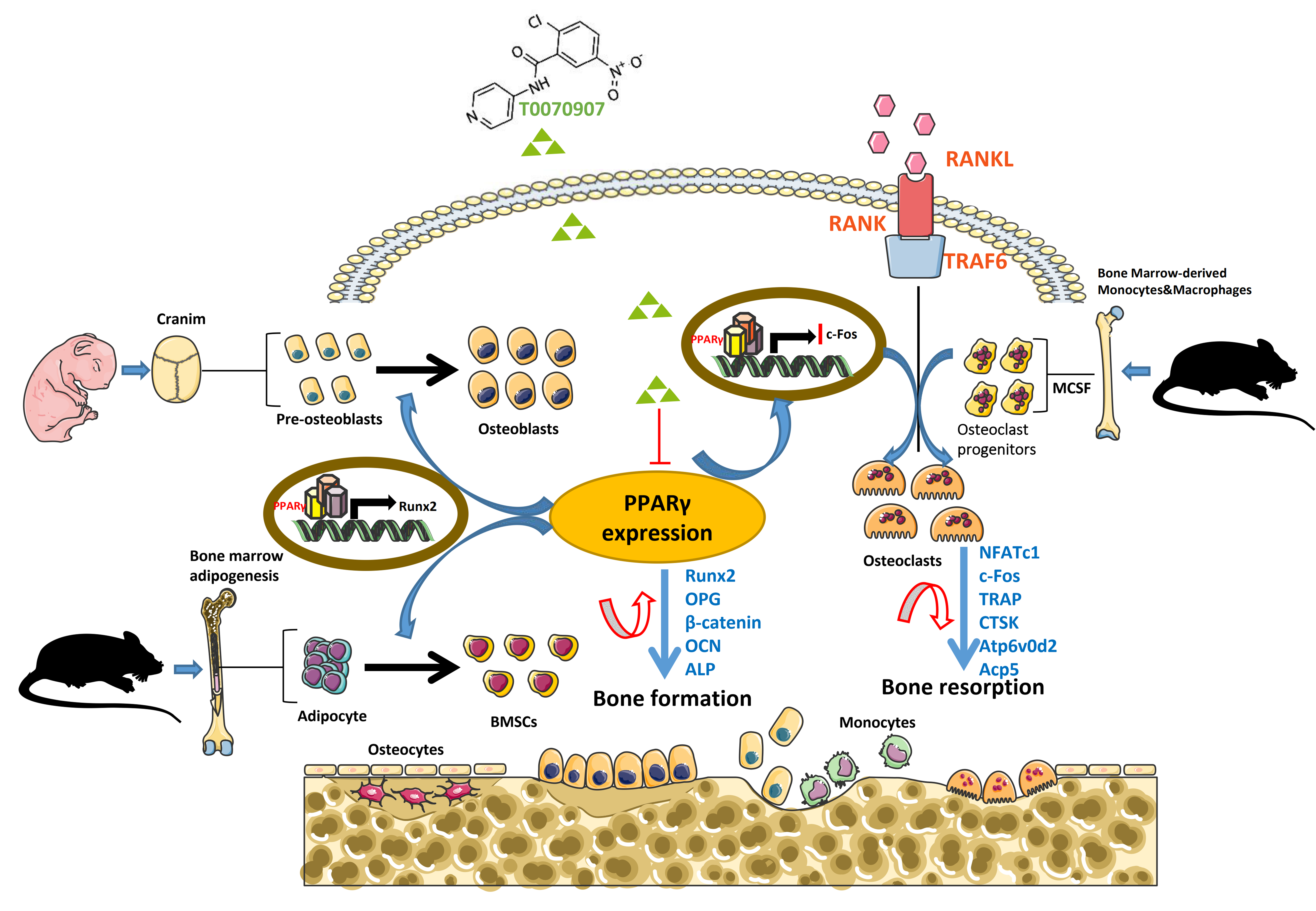

Supplement: Supplementary file 2 — Additional file 2: Figure S2. A simplified model of the mechanism by which T007 inhibits osteoclastogenesis and osteoblastogenesis. T007 reduces PPARγ expression that inhibits osteoclast progenitor’s differentiation into osteoclasts, thus attenuating bone resorption. In osteoclast progenitor, T007 suppress c-Fos expression, which stimulates osteoclastogenesis. Conversely, T007 restrains PPARγ activation that promotes pre-osteoblasts differentiation into osteoblasts and contributes to BMSCs growth by increasing Runx2 expression, thus enhancing bone formation. Consequently, T007 results in bone loss by tipping in balance of bone remodeling through concerned stimulation of bone resorption and inhibition of bone formation. [file 12964_2019_442_MOESM2_ESM.tif]
